# Body of Evidence in Favor of Adopting 130/80 mm Hg as New Blood Pressure Cut-Off for All the Hypertensive Disorders of Pregnancy

**DOI:** 10.3390/medicina55100703

**Published:** 2019-10-20

**Authors:** Giovanni Sisti, Belinda Williams

**Affiliations:** 1 Department of Obstetrics and Gynecology, Lincoln Medical and Mental Health Center, Bronx, NY 10451, USA; 2 St. George’s University School of Medicine, True Blue, West Indies, Grenada; belindawilliams19@gmail.com

**Keywords:** hypertensive disorders of pregnancy, blood pressure cut-off, preeclampsia, gestational hypertension, eclampsia

## Abstract

The American College of Cardiology/American Heart Association (ACC/AHA) updated its guideline redefining the classification of hypertension and the blood pressure cut-off in 2017. The current cut-offs for stage 1 hypertension of 130 mm Hg systolic blood pressure or 80 mm Hg diastolic blood pressure replace the previous cut-offs of 140 mm Hg systolic blood pressure or 90 mm Hg diastolic blood pressure which were based on the ACC/AHA guidelines from 1988. However, the blood pressure cut-off for the obstetric population still remains as 140/90 mm Hg despite the scarcity of evidence for it. Recent American College of Obstetricians and Gynecologists (ACOG) bulletins for pregnant women have not reflected the new ACC/AHA change of guideline. We reviewed a mounting body of evidence prompting the implementation of the new ACC/AHA guidelines for the obstetric population. These studies examined maternal and fetal outcomes applying the new ACC/AHA guidelines during antepartum or postpartum care.

## 1. Introduction

Hypertensive disorders of pregnancy (HDP) occur in 10% of pregnancies in women of reproductive age globally [1]. As such, they constitute one of the leading causes of maternal and perinatal mortality worldwide. HDP are classified as chronic hypertension (CHTN), gestational hypertension (GHTN), preeclampsia, and eclampsia. The blood pressure cut-off for these disorders has been established by the 2019 American College of Obstetricians and Gynecologists (ACOG) issued practice bulletins Number 202 titled, “Gestational Hypertension and Preeclampsia” and Number 203 titled, “Chronic Hypertension in Pregnancy”, which update and replace the ACOG-Hypertension in Pregnancy task force report developed in 2013.

**CHTN** in pregnancy is present in 0.9–1.5% of pregnant women. It is defined as hypertension diagnosed or present before pregnancy or before 20 weeks of gestation. The cut-off is systolic blood pressure of 140 mm Hg or more, or diastolic blood pressure of 90 mm Hg or more. It is associated with poor perinatal outcomes including low birth weight, preterm delivery, higher perinatal mortality, and an increased risk for neonatal death.

**GHTN** is defined as systolic blood pressure of 140 mm Hg or more, or diastolic blood pressure of 90 mm Hg or more after 20 weeks of gestation, and its prevalence has been estimated to be 1.8–4.4% of the pregnancies [2,3]. The blood pressure measurements have to be made at two different time points at least 4 h apart. There is no proteinuria or other systemic findings, such as headache or abdominal pain. While maternal and perinatal outcomes of GHTN are generally less severe, about 50% of women with GHTN can progress to preeclampsia. It may also be followed by long-term CHTN.

**Preeclampsia** complicates 2–8% of pregnancies globally [4]. Preeclampsia occurs when blood pressure is greater than 140/90 mm Hg after 20 weeks of gestation. It is accompanied by proteinuria, or signs/symptoms like right upper quadrant pain, persistent epigastric pain, severe headache, blurry vision, thrombocytopenia, elevated liver enzymes, renal insufficiency and pulmonary edema [2]. Uteroplacental ischemia in preeclampsia results in fetal growth restriction and oligohydramnios. Maternal consequences of the condition include placental abruption and increased risk of preterm delivery.

**Eclampsia** occurs in about 1% of pregnancies [5]. It is one of the most severe manifestations of all HDP and is characterized by new-onset seizures in a pregnant woman with no history of epilepsy or seizure-provoking neurological conditions or drug use [6]. Eclampsia is associated with increase in neonatal and infant morbidity and mortality, including fetal demise, intrauterine growth restriction, preterm birth, respiratory failure, neonatal thrombocytopenia, and bronchopulmonary dysplasia. The majority of maternal deaths for HDP occur in the **postpartum** period, up to 42 days after delivery [7,8]. As a result, ACOG recommends monitoring blood pressure in the hospital or equivalent outpatient surveillance for at least 72 h postpartum with a follow-up blood pressure check 7–10 days later.

We have previously shown that the usage of the 140/90 mm Hg cut-off is affected by several flaws: it is not evidence-based, it is established upon the Joint National Committee (JNC) report guideline set in 1988 and does not reflect the recent American College of Cardiology/American Heart Association (ACC-AHA) task force guideline [9,10].

In 2017, the ACC-AHA task force on clinical practice changed the guideline for the diagnosis of stage 1 hypertension, the blood pressure cut-off changed from ≥ 140/90 mm Hg to ≥ 130/80 mm Hg. In developing this recommendation, the writing committee drew upon evidence-based methodologies. Literature searches focused on randomized controlled trials, registries, non-randomized comparative and descriptive studies, case series, cohort studies, systematic reviews, and expert opinions. The current guideline from 2017 has updated and replaced the prior Joint National Committee (JNC) guideline from 1988.

In fact, among the two latest ACOG bulletins previously mentioned about HDP, only one of them, Number 202 (on CHTN), mentions the new ACC/AHA guidelines. In the ACOG bulletin Number 202, the new ACC/AHA guidelines are still considered an active area of investigation when applied to pregnant women as it may mislabel some women as abnormal.

However, there is a growing body of evidence accumulating against the usage of 140/90 mm Hg and in favor of the incorporation of the new 130/80 mm Hg cut-off into the new guidelines. Given the ethical concerns associated with running clinical trials on pregnant patients in the third trimester, all the studies published to date are retrospective, and they all attempt to answer the question—what would happen to the maternal and fetal outcomes if 130/80 mm Hg instead of 140/90 mm Hg would had been used to diagnose HDP?

Hereby, we want to summarize the current evidence in favor of lowering the threshold for HDP from 140/90 mm Hg to 130/80 mm Hg (see Table 1).

## 2. Studies That Examined the Patients at <20 w

Hauspurg et al. in 2019 utilized data from the Nulliparous Pregnancy Outcomes Study: Monitor Mothers-to-Be cohort. They performed a secondary analysis of 8924 patients recruited between 2010–2014 in eight clinical sites in the US [11]. Patients with a history of CHTN (blood pressure ≥ 140/90 mm Hg) and diabetes were excluded. The researchers discovered that the development of stage I hypertension in the first trimester, with the new ACC/AHA criteria, was statistically significantly associated with subsequent preeclampsia, GHTN, gestational diabetes mellitus (GDM), preterm birth, birth weight, and operative vaginal delivery.

Sutton et al. in 2018 conducted a secondary analysis of data from a multicenter randomized, double-blind, placebo-controlled trial of low-dose aspirin for prevention of preeclampsia in nulliparous, low-risk women recruited between 13 and 25 weeks of gestation [12]. They excluded patients with CHTN (blood pressure > 135/85 mm Hg), renal disease or proteinuria. In the placebo arm consisting of women recruited before 20 weeks of gestation, they found a stastistically significant association between stage I hypertension with the new ACC/AHA criteria (systolic 130–135 mm Hg and diastolic 80–85 mm Hg) measured before 20 weeks and the risk of preeclampsia, GDM, and small for gestational age later on during pregnancy.

## 3. Studies That Examined Patient at >20 w

Hu et al. in 2019 assessed the impact of the 2017 ACC/AHA hypertension guidelines on the diagnosis of GHTN and associated maternal and neonatal risks [13]. They used data from a birth cohort of 16,345 women without CHTN who delivered at the Wuhan Women and Children Medical Care Center in Wuhan, China, from 2012 to 2016. GHTN diagnosed after 20 weeks of gestation was significantly associated with adverse perinatal outcomes, including preterm birth and small for gestational age.

Fukushima et al. in 2012 investigated the relationship between a pre-hypertension range blood pressure after 32 weeks of gestation and fetal growth. Patients were recruited in Japan from 2008 to 2010 [14]. Their exclusion criteria specified any diagnosis of hypertension or preeclampsia, gestational or pre-existing diabetes mellitus, thyroid disorders, active collagen disease, liver or renal dysfunction, placenta previa, uterine or fetal malformations during pregnancy. Patients were also excluded for use of alcohol or tobacco during pregnancy. Pre-hypertension was considered having diastolic blood pressure 80–89 mm Hg and systolic blood pressure 130–139 mm Hg. There were statistically significant differences in the ratio of neonatal birth weight and the deviation from standard birth weight and the percentage of small for gestational age in the control and the pre-hypertension group.

## 4. A Study That Examined Patients in the Postpartum Period

Smith et al. applied the new ACC/AHA criteria for hypertension to two existing postpartum databases to determine what proportion of women who had an uncomplicated pregnancy or a pregnancy complicated by a hypertensive disorder, would now be classified with a diagnosis of hypertension [15]. One cohort was analyzed at six months and the other cohort at 12 months. The recruitment periods for both the databases together ranged from 2003 to 2017. The patients were considered to have stage I hypertension if the diastolic blood pressure was 80–89 mm Hg or systolic blood pressure was 130–139 mm Hg. In the control group, 22% would have been classified as stage I hypertension instead of < 1% using the old criteria. For patients with HDP during pregnancy, the new values stratified 56.4% and 67.2% of women as stage I hypertension, compared to 20.9% and 31.3% using the old criteria, in the two cohorts, respectively.

In summary, the usage of the new criteria for the diagnosis of high blood pressure in the postpartum period would be labeled as affected by “postpartum HDP” in more than twice the number of patients identified by the old criteria.

## 5. Discussion

The evidence against the continuation of the old 140/190 mm Hg criteria and in favor of the usage of the new 130/80 mm Hg criteria is definitely growing. Two studies analyzed the effect of the new criteria when applied in the first trimester or early second trimester, another two focused on the diagnosis of HDP with the new criteria after 20 weeks of gestation, and one examined the implications of the change in criteria in the postpartum period.

All the studies were retrospective. The two studies analyzing pregnancies before 20 weeks selected nulliparous women, while the ones focusing on later stages of pregnancy included nulliparous and multiparous women. The population region of origin varied from China and Japan to the USA.

The selection criteria and the analyzed outcomes were heterogeneous, but they all reached significant associations between blood pressure higher than 130/80 mm Hg and increased maternal and neonatal mortality. In particular, they found an increased prevalence of preeclampsia, GHTN, GDM, preterm birth, operative vaginal delivery, and small for gestational age.

## 6. Conclusions

Although evidence emerging from the aforementioned studies is increasing, it remains weak. All the available studies are retrospective in nature owing to the intrinsic ethical concerns associated with the pregnant status. In addition, there are no studies analyzing the diagnosis of preeclampsia itself, because in patients with blood pressure below 140/90 mm Hg, we do not measure proteinuria on a routine basis.

However, this is the same level of evidence that we had when the current guidelines were written. Given the different maternal and fetal outcomes with the adoption of the new ACC/AHA guidelines, we encourage rewriting new guidelines for the obstetric population, using these updated research findings.

We hereby propose the implementation of the new ACC/AHA criteria to the diagnosis of HDP.

## Figures and Tables

**Table 1:** Studies analyzing maternal and fetal outcomes after applying the new blood pressure cut-off proposed by ACC/AHA for diagnosis of hypertension.

Authors	Year of Publication	Years of Cohort Analysis	Study Design	BP Cut-Off	Exclusion Criteria	Parity	GA of BP Measurement	Number of Patients Included	Fetal Outcome	Interventions
Hauspurg A. et al.	2019	2010–2014	Secondary analysis from a prospective observational study	Systolic 130–139 mmHg or diastolic 80–89 mmHg	CHTN ≥ 140/90, and pregestational diabetes	Nulliparous	12.1 ± 1.5 weeks, enrollment at 6–13.6 weeks	8924	Significantly associated with subsequent preeclampsia and gestational HTN, GDM, indicated PTB, birth weight, operative vaginal delivery	Not studied
Sutton E. et al.	2018	1989–1992	Secondary analysis of data collected in a randomized, double-blind, placebo-controlled trial	Systolic 130–135 mmHg or diastolic 80–85 mmHg	CHTN > 135/85 mmHg, renal disease, proteinuria	Nulliparous	<20 weeks	1661	In the placebo arm: preeclampsia; GDM, SGA (<10%)	ASA was not associated with poor fetal outcomes
Hu J. et al.	2019	2012–2016	Retrospective cohort	Systolic 130–139 mmHg and diastolic 80–89 mmHg	CHTN ≥ 130/80	Not specified	>20 weeks, within one month before delivery	3422	Significantly increased risk of SGA and PTB	Not studied
Fukushima et al.	2012	2008–2010	Retrospective cohort	Systolic 130–139 mmHg and diastolic 80–89 mmHg	GHTN, preeclampsia, GDM, pregestational DM, thyroid disorders, active collagen disease, liver or renal dysfunction, placenta previa, uterine/fetal malformations	54.9% nullipara	>32 weeks	71	SGA	Not studied
Smith et al.	2019	PE-NET: 2003–2010, MHC: 2011–2017	Two Retrospective cohorts	Systolic 130–139 mmHg or diastolic 80–89 mmHg	HTN, pregestational DM, GDM, cardiovascular disease, Renal disease, women living outside a 50 km radius from the study hospitals	Not specified	Not specified	505 (PE-NET 240, MHC 265)	N/A	N/A (postpartum analysis)

CHTN = Chronic Hypertension, GHTN = Gestational Hypertension, GDM = Gestational Diabetes Mellitus, Cohorts PE-NET = Pre Eclampsia-New Emerging Team, MHC = Maternal Health Clinic, GA = Gestational Age, BP = Blood Pressure, PTB = PreTerm Birth, SGA = Small for Gestational Age, N/A = Not Applicable.
